# The intersection of biology and advanced technologies defines the future of dairy reproductive management

**DOI:** 10.3168/jdsc.2025-0840

**Published:** 2025-09-25

**Authors:** M.C. Lucy

**Affiliations:** Division of Animal Sciences, University of Missouri, Columbia, MO 65211

## Abstract

Advances in reproductive management in dairy cattle have typically been made through the application of new technology. The past 25 yr have been a period of rapid improvement in dairy reproduction through the implementation of new technology that was unknown a short time ago. The next 25 yr will likely be similar with the refinement of existing technology and development of new technology to address the current challenges to dairy reproduction. Many of these new developments will address the need for greater on-farm automation necessitated by the consolidation of farms (more cows per farm) in the face of shortages in large animal veterinary and agricultural labor. Genomics will continue to play an important role in improving fertility on farm. There is the possibility that postpartum uterine health could benefit from advanced technologies that are emerging as effective treatments for tissue damage caused by disease. The reprogramming of somatic cells into gametes (in vitro gametogenesis) or embryos (blastoids) may entirely change the methods used to propagate elite genetics from female animals. Despite important advances in new technology, there are also important questions and researchable topics that need to be addressed so that the field of dairy reproduction can advance. These include automating or simplifying the current method of artificial insemination, addressing the short lifespan of sperm in the reproductive tract, and solving fertilization failure and embryonic loss following insemination. Embryo technologies await new discoveries to improve embryo yield from donor animals, increase the development of embryos in culture, reduce the damage caused by freezing embryos, and effectively evaluate embryo quality before transfer. The collective result of new knowledge and new technology will drive further improvements in genetics, fertility, and the efficiency of reproduction in high-producing dairy cattle.

## Highlights

• New technology will address current challenges to dairy reproduction.
• Consolidation (larger farms) will lead to a greater reliance on automation.
• Improvements will be made to existing technologies.
• Emerging technologies have the potential to effect major changes.
• Important questions and researchable topics need to be addressed.

## Graphical Abstract

**Figure:**
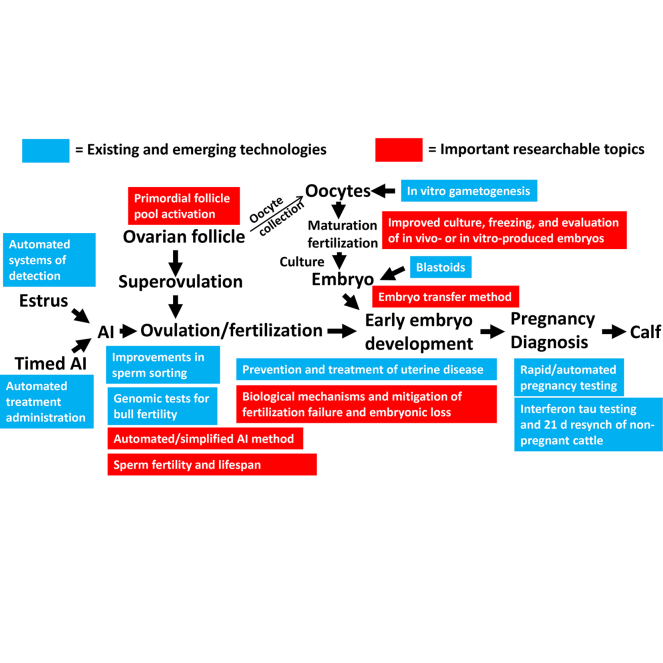
**Summary:** The past 25 years have been a period of rapid improvement in dairy reproduction through the implementation of new technologies that were unknown a short time ago. The next 25 years will likely be similar, with the refinement of existing technology and the development of new technology to address current challenges to dairy reproduction. Many of these new developments will address the need for greater on-farm automation, necessitated by the consolidation of farms (more cows per farm) in the face of shortages in large animal veterinary and agricultural labor. Despite important advances in new technology, there are also important questions and researchable topics that need to be addressed so the field of dairy reproduction can advance. The collective result of new knowledge and new technology will drive further improvements in genetics, fertility, and the efficiency of reproduction in high-producing dairy cattle.

A lot has changed in dairy reproductive management since the publication of “Reproductive loss in high-producing dairy cattle: Where will it end?” 25 yr ago (Lucy, 2001). Examples of reproductive technologies that were not routinely practiced or known at the time of the 2001 publication include Ovsynch and its subsequent iterations (Presynch, Ovsynch, double Ovsynch, Resynch, and so on; Fricke and Wiltbank, 2022); genomics and genomic selection for reproductive traits (Lucy, 2019); sex selection of spermatozoa (Seidel, 2014); and pregnancy diagnosis using blood or milk (Ott et al., 2025). These new technologies were introduced and expanded into the dairy industry to reverse the negative reproductive trends that existed in the early 2000s. The present-day dairy industry faces a new set of challenges to efficient reproduction that include larger herds and fewer agricultural workers per farm (Lucy and Pohler, 2025). The next 25 yr in bovine reproduction will inevitably prove to be equally remarkable to the previous 25 yr with respect to the technologies that are developed and implemented on farm. In 1981, Dr. Thomas E. Starzl, the father of organ transplantation, wrote “what was inconceivable yesterday, and barely achievable today, often becomes routine tomorrow” (Eghtesad and Fung, 2017, p. e2). The thoughts of Dr. Starzl are clearly applicable to the reproductive technologies used on dairy farms. The objective of this narrative review is to assess the present-day status of reproductive research and associated technologies on dairies and highlight the “barely achievable” technologies that may become “routine” on tomorrow's farms. Existing and emerging technologies (Table 1), as well as research topics for the future (Table 2), are presented.

**Table 1:** Expectations for existing and emerging technologies to increase reproductive success in dairy cattle

Existing technologies	Emerging technologies
• Greater sensitivity and specificity for estrus detection systems based on activity, location, and novel behaviors indicative of estrus. • Alternative methods for the administration of PGF_2α_ and GnRH treatments in timed AI systems including robotic arms, needle-free systems, and automated intravaginal delivery. • Further improvements in the sorting efficiency and fertility of sex-selected semen. • Development of highly sensitive genomic tests to predict field fertility of AI sires • Automation of in-parlor milk sampling and analysis for the purpose of pregnancy diagnosis based on milk pregnancy-associated glycoproteins (PAG). • Further development of methods to detect pregnancy based on interferon tau (IFNT) to enable resynchronization of estrus in nonpregnant cows within 21 d after breeding.	• Development of biomarkers in milk (including LH) that complement milk progesterone for estrus detection. • Milk or blood pregnancy testing that competes directly with ultrasound for instantaneous pregnancy diagnosis on farm. • Prevention and treatment of fibrosis and tissue damage caused by infection and uterine inflammation postpartum. • Reprograming damaged uterine tissue using chemical reprograming factors. • In vitro gametogenesis (derivation of fertile oocytes from induced pluripotent stem cells for in vitro fertilization and the production of embryos for transfer). • Blastoids (derivation of large numbers of fertile blastocysts from induced pluripotent stem cells)

**Table 2:** Researchable topics with important long-term implications for dairy reproduction

Researchable topics
• Automated or simplified methods for AI. • Factors that affect sperm lifespan in the female reproductive tract and strategies that can be used to increase sperm lifespan to enhance fertility. • Biological mechanisms and mitigation of fertilization failure and embryonic loss following AI. • Mechanisms to stimulate the development of primordial to primary follicles and sustain development to the antral follicle stage for the purpose of improving the number of harvestable oocytes and embryos from individual cattle. • Improvements in the culture of bovine embryos and cryopreservation of bovine embryos and gametes (oocytes and spermatozoa). • New methods to identify high-fertility bovine embryos before transfer and refinements to the transcervical embryo transfer technique to increase the survival of implanted embryos.

The current focus for estrus detection is the development of more sensitive wearable electronic technology. The primary means of detection is an accelerometer and in some cases location sensors that measure animal movement (Cerri et al., 2021). The addition of a rumination sensor improved the sensitivity of detection because cows in estrus eat and ruminate less (Beauchemin, 2018). There is also the possibility of the detection of estrus through body temperature monitoring (rumen bolus, ear tag, or vaginal implant; Reith and Hoy, 2018). Remote systems for estrus detection have the added benefit of detecting sick cows that also eat and ruminate less, are less active, and have elevated body temperature. Wearable devices fit into the need for greater on-farm automation to address a reduced agricultural labor force (Lucy and Pohler, 2025). Increased sensitivity will likely be achieved through an improved understanding of estrus behavior (movements, social interactions, and so on) and the collection of additional data on individual cows that may not be linked to their movement or rumination (Cerri et al., 2021). It is very likely that data (health, reproduction, milk production, BCS, and so on) from wearable and other remote sensing devices as well as genomic testing will be combined so that health and reproductive programs are tailored to the benefit of fertility in the individual cow (“targeted reproductive management”; Giordano et al., 2022; Bruinjé and LeBlanc, 2024; Chebel et al., 2025).

The onset of estrus coincides with the LH surge that causes ovulation ∼30 h later (De Rensis et al., 2024). If the time of the LH surge is known, then the time of ovulation is known and the detection of estrus is not necessary. The Herd Navigator system (DeLaval, Tumba, Sweden) is an inline milk progesterone monitoring technology consisting of a “biomodel” that signals the milking robot for sample collection from individual cows, an autosampler (for milk collection), and a progesterone analysis unit that measures milk progesterone (Bruinjé et al., 2025). One limitation of the system is that the time of estrus can only be approximated based on the time of luteolysis. This may be explained by the high variability in circulating estradiol before estrus caused by hepatic metabolism of estradiol in high-producing dairy cows (Wiltbank et al., 2011). Using a wearable device for the detection of estrus as well as the Herd Navigator system (milk progesterone analysis) would greatly increase the sensitivity of the estrus detection because false-positive cows (high activity with high progesterone) could be identified and not inseminated. Developing a module for LH surge detection in milk or alternative milk biomarkers for estrus could also be used to pinpoint the ideal time for breeding. Inline near-infrared analysis of milk samples in real time (during milking) could provide an alternative means of detecting reproductive hormones or other reproductive biomarkers in lactating cows (Giannuzzi et al., 2022).

Timed artificial insemination (**AI**) programs circumvented the need for estrus detection (Fricke and Wiltbank, 2022). Most dairies in the United States rely entirely or in part on timed AI programs to achieve acceptable reproductive rates and create the “high fertility cycle” where cows are inseminated and become pregnant at a targeted postpartum interval that is appropriate for the dairy (Fricke et al., 2023). Other than the ethical concerns of repeated intramuscular injections expressed by some and unfounded human food-safety concerns, the only limitation of the timed AI technology is the labor required to identify and treat cows. Using self-locking head locks to “lock up” an entire pen of cows to find and treat a small number of cows reduces milk production of the pen compared with control (Paudyal et al., 2023). Electronic means to identify cows needing treatment as they move through the milking parlor or sort gates in the parlor return alley (or both) provide a means to separate cows efficiently for treatment. Installation of a robotic arm for treatment administration would further reduce the need for farm labor. Needle-free systems could simplify treatment administration and reduce animal stress response (Leslie et al., 2025). The development of a programmable intravaginal device for the delivery of PGF_2α_ and GnRH treatments holds promise to completely automate timed AI programs (Ren et al., 2023). In the meantime, dairies are increasingly combining timed AI with automated estrus detection systems to identify cows in estrus before timed AI and before pregnancy diagnosis to reduce the labor and expense associated with timed AI treatments (Laplacette et al., 2024).

The cervix separates the vagina from the uterine body. This important biological barrier protects the uterine lumen from bacterial or viral infection (Miller, 2024). The cervix also represents the greatest barrier to efficient insemination of cattle that is not easily solved through automation or overcome with new technology. Artificial insemination as it is practiced today is essentially the same as it was practiced over 75 yr ago (Salisbury and Vandemark, 1951). The author is not aware of any new technology designed to automate the transcervical AI method. A novel method of semen administration with acceptable fertility would be a welcome innovation to the dairy industry.

The development and implementation of AI as a tool to improve the genetics of dairy cows was revolutionary technology that was enabled by the dilution and storage of semen. Sorting of bovine spermatozoa into X- and Y-chromosome-bearing fractions created a second revolution enabled by new technology (Seidel, 2014). Although progress has been made, preservation techniques for spermatozoa are imperfect with ∼50% of cells dying during freezing/thawing and there are inconsistent and unexplained responses to cryopreservation for individual sires (Yánez-Ortiz et al., 2022). These inconsistencies lead to considerable variation in the fertility of sires that ultimately affect the success of AI. “Fresh” semen (not cryopreserved) is routinely used in grass-based dairy production systems because of high semen demand during the breeding season. Fresh semen is loaded at a lower sperm dose per straw (compared with frozen) and can achieve equal fertility. The number of inseminations from a single ejaculate, therefore, can be increased if fresh semen is used (Murphy et al., 2016). Fresh sex-sorted semen loaded at 1 × 10^6^ sperm per straw had similar fertility to conventionally frozen sex-sorted semen loaded at 2 × 10^6^ sperm per straw in both cows and heifers (Maicas et al., 2019). The use of fresh sex-sorted semen instead of cryopreserved sex-sorted semen may further extend the salable units of sexed semen from a single ejaculate and simplify the insemination procedure by eliminating the thawing step.

The task of creating high-fertility sires is made difficult by the fact that the traits of female fertility (typically included in selection indices) and male fertility are not highly correlated. Recent progress in identifying genomic markers for male fertility may hold promise for the identification of high-fertility sires at the embryonic stage when genomic tests are typically done (Peñagaricano, 2024).

Cattle are outliers among animal species with respect to survival of spermatozoa in the reproductive tract (∼24 h) with a more typical survival time among species being measured in multiples of days, months, or years (Holt, 2011). If the survival of spermatozoa in the bovine reproductive tract were longer, then the timing of AI could be less precise, perhaps enabling a single insemination several days before ovulation with acceptable fertility. Sperm microencapsulation may extend the lifespan of sperm but has not achieved fertility superior to conventional insemination (Nebel and Saacke, 1994). The fertility of a sire depends, in part, on the lifespan of their sperm in the female tract (Saacke et al., 2000). Identifying the biological mechanisms of sperm survival in the female tract and interceding to increase the sperm lifespan holds great promise for increasing fertility and simplifying reproductive management on farm (Miller, 2024).

Recent studies have demonstrated that the period from ovulation to the entry of the fertilized embryo into the uterus (morula stage embryo; 5 to 7 d after AI) is the time of greatest embryonic loss (Berg et al., 2022; Crowe et al., 2025). The losses during this time (30%) are approximately split between fertilization failure and subsequent developmental failure. Neither fertilization failure nor developmental failure can be satisfactorily explained based on existing knowledge but are entirely consistent with in vitro fertilization where a percentage of oocytes do not fertilize and many fertilized embryos do not develop (Moore and Hasler, 2017). An additional 10% to 15% of embryos die while the embryos develop to the filamentous stage and leading up to maternal recognition of pregnancy. The collective loss from the 2 initial periods of development (fertilization to the initiation of maternal recognition of pregnancy) is 40% and there is minimal understanding of why embryos are lost during this period and no effective strategies to mitigate this loss. The failure to address this loss is in part explained by our inability to easily identify cows that are pregnant or not pregnant during this early period, and this makes the investigation of the associative causes of the embryonic loss difficult to study.

The secretion of interferon tau (**IFNT**) from the embryo as the maternal recognition of pregnancy signal (Johnson et al., 2024) and the formation of placental binucleate cells that secrete pregnancy-associated glycoproteins (**PAG**; Ott et al., 2025) initiate a period where pregnancy can be detected using a chemical test. The PAG provide a slightly earlier result (∼28 d after AI) when compared with ultrasound for pregnancy diagnosis that is typically done 32 d after AI. The chemical testing of PAG does not require the skilled labor that is needed for an ultrasound exam. Sample collection and the downstream analyses can be performed on the farm using unskilled labor and the sample analyzed on farm or at central laboratories. The disadvantages of PAG (compared with ultrasound examination) are (1) a PAG test does not provide a cow-side (instantaneous) pregnancy diagnosis that would allow the immediate treatment of nonpregnant cows (typically a PGF_2α_ injection); (2) a PAG test cannot differentiate between a healthy conceptus (with heartbeat) and dead conceptus (present but without a heartbeat); and (3) there is also a long half-life of PAG in the circulation of cows that have undergone embryonic loss (Giordano et al., 2012). Lateral flow tests for PAG provide pregnancy results within 15 to 30 min (marketed by IDEXX [Westbrook, ME] and BioTRACKING [Moscow, ID]). Instantaneous chemical diagnosis of pregnancy like that achieved with a handheld glucose monitor (5 s from sample application to result) is a challenging goal that would require a novel platform for small molecule analysis. Blood sample collection is difficult to automate on farm, but automated milk sampling into a bar-coded vial for individual cows is easily achievable technology. Regardless of the test speed, the ease of automation for milk sampling will likely make PAG milk testing the primary means of pregnancy diagnosis on future dairies.

Measuring IFNT is difficult in blood but circulating leukocytes can be assayed for interferon stimulated genes (**ISG**) including *OAS1*, *MX2*, and *ISG15* (Ott et al., 2025). Sampling cervical or vaginal cells (in lieu of peripheral blood) demonstrated that tissues near the pregnancy have both IFNT and a robust ISG response. Bishop et al. (2025) reported the development of a cervical sampling device and an IFNT ELISA assay that can be used to diagnose pregnancy within 16 d after AI (Bishop et al., 2025). This system allows pregnancy diagnosis and resynchronization of nonpregnant cows before the “natural” return to estrus, which has distinct advantages in dairy production systems (Lucy, 2025).

The final period of major embryonic loss (28 to 60 d of pregnancy) has received considerable attention because the embryonic loss can be measured throughout this period using a combination of chemical pregnancy tests and ultrasound examination (Binelli et al., 2025). Not unexpectedly, the proposed causes of embryonic loss are diverse. Santos et al. (2009) concluded that “factors that reduced conception rate on day 30 after AI also increased pregnancy loss between 30 and 58 d of gestation” (Santos et al., 2009, p. 207). Whatever factors reduce pregnancies per AI, therefore, do not appear to selectively target different stages of pregnancy up to d 60 when the placenta is fully formed.

Cows with early postpartum uterine disease have lesser fertility later in lactation. This paradigm is similar to that proposed in the “Britt hypothesis” (Britt, 1991). According to the Britt hypothesis, oocytes that initiate their development early postpartum during negative energy balance are damaged and ovulate during the breeding period with a lower fertility. Likewise, uterine disease early postpartum (metritis and endometritis) damages the uterus so that uterine function is compromised (Caldeira et al., 2025) and fertility during the breeding period is lower (∼20 percentage points lesser pregnancy per AI at first postpartum AI; Bruinjé et al., 2024). Some of the uterine “damage” is easily recognized including the presence of inflammation and lymphocytic foci (Lucy et al., 2016) as well as adenomyosis and fibrosis (Sellmer Ramos et al., 2025). Fibrosis is typically viewed as an inevitable outcome of tissue damage, but recent studies in humans suggested otherwise where fibrosis (scar tissue formation) can be prevented with the antimalarial drug artesunate (Zhang et al., 2024) or treated with CAR-T cells (Zhang et al., 2025).

“Inflammatory memory” is the process through which inflammation imprints nonimmune cells within the affected tissue to permanently change their function (Naik and Fuchs, 2022). We found evidence for this in a recent study where the uterine transcriptome of cows that were previously healthy or metritis were compared following slaughter ∼80 or 165 d postpartum (Silva et al., 2024). The analysis identified a large effect of early postpartum metritis on the uterine transcriptome later postpartum. The metritis had a greater effect on the caruncular endometrium (uterine sites for placental attachment) compared with the intercaruncular endometrium. This specific effect on the caruncular endometrium may explain why placentation fails in cows that previously had metritis. To restore full fertility, it may be necessary to “partially reprogram” uterine cells meaning that epigenetic imprints created by disease are erased so that cells return to a naïve state (i.e., their baseline epigenetic state that was present before being exposed to the inflammatory milieu created by the immune response to uterine disease). Reprograming of cells was traditionally done by transfecting cells with transcription factor genes (*Oct4*, *Sosc2*, *Klf4*, and *Myc*; OSKM; “Yamanaka factors”) that were stably integrated into the genome of the host cells. The stable expression of these genes reprogrammed cells to a more stem-like state (Yücel and Gladyshev, 2024). “Partial chemical reprograming” is similar but a chemical cocktail is used in lieu of the OSKM factors (thus avoiding genetic modification of host cells). The partial reprogramming can reverse aging and erase epigenetic marks associated with disease (Cipriano et al., 2024). The new program could restore fertility to an otherwise damaged and infertile uterus.

Genomics will continue to play a large part in the reproduction of future cows. Most modern-day selection indices have a heavy weighting on health, longevity, and fertility. A look into the future is provided by the “Next Gen” study at Teagasc Moorepark (Ireland). In a study that encompassed 5 lactations, the Irish Economic Breeding Index (**EBI**) was tested by comparing cows within the top 5% nationally for the EBI (elite cows) versus cows with the national average (**NA**) EBI (based on genomic testing). The EBI is heavily weighted for fertility (∼25%) because seasonal calving herds in Ireland have a requirement for a 1-yr calving interval. Although milk solids production was marginally affected (elite slightly greater than NA), the fertility and survival was greater for elite versus NA with ∼60% of elite cows remaining in the herd to 5 lactations compared with ∼40% for NA cows. The elite cows had greater BCS and improved metabolic indicators (greater glucose, insulin, and IGF1 and reduced BHB; O'Sullivan et al., 2020). The “stress” of milk production was the same for the 2 groups because the production was almost identical, but the “strain” created by the production (based on glucose, insulin, IGF1, and BHB) was less in the elite. Traditional dairy selection indices with a heavy weighting on milk production created considerable strain through negative energy balance and BCS loss in early lactation (Lucy, 2019). Modern genetic selection indices that achieve similar milk production with less BCS loss and normalized metabolic indicators will benefit the reproduction of future cows.

Superior dairy genetics is generally disseminated through elite sires with a superior genomic test. Compared with males, the superior genetics found in an individual female is vastly more difficult to propagate. Although superovulation or transvaginal oocyte recovery/in vitro embryo production can be applied to females, these procedures have only marginally improved with respect to embryo yield since their inception over 60 yr ago (Moore and Hasler, 2017). Mammals are programed to maintain follicles in a quiescent primordial pool that are released incrementally over time into the growing pool (thus preserving the nonrenewable pool of gametes throughout the reproductive lifespan; Zhao et al., 2021). The failure to improve embryo yield traces back to the yet unsolved problem of how to release a greater number of primordial follicles from the quiescent pool (Picton, 2022). The phosphatidylinositol 3-kinase (PI3K) growth factor pathway is clearly involved in primordial follicle pool activation because oocyte-specific deletion of *Pten* (a negative regulator of the PI3K pathway) led to the activation of primordial follicles and complete depletion of the primordial follicle pool (Reddy et al., 2008). The PI3K pathway is activated by both insulin and IGF1, both of which are closely tied to ovarian follicular development in cattle (Lucy, 2000). Ovarian inactivation of *PTEN* and the large-scale activation of primordial follicles in cattle, however, has not been achieved.

Progress has been made with respect to superovulation as well as the in vitro maturation and fertilization of oocytes and the culture and freezing/thawing of embryos (Moore and Hasler, 2017). Nonetheless, the technology requires considerable expertise and the efficiency of embryo production is low (∼25% of oocytes collected yield transferable embryos). Embryonic loss was greater for a frozen compared with a fresh embryo transfer indicating that current methods of freezing and thawing will damage the embryo (Crowe et al., 2025). Inconsistent responses among donor cows submitted to identical superovulation protocols continue to frustrate practitioners (Moore and Hasler, 2017).

Crowe et al. (2025) demonstrated that the embryonic losses through d 18 were identical for AI and fresh embryo transfer. Hansen (2020) raised an important question relative to embryo transfer in cattle. Specifically, if embryonic loss rates during the first week after breeding are ∼30% then why doesn't the transfer of a high-quality embryo after d 7 (thus circumventing the embryonic loss before d 7) have a greater pregnancy rate when compared with AI? The observation that embryo transfer does not yield increased fertility relative to AI suggests that either the transcervical embryo transfer kills a percentage of embryos or an embryo that appears morphologically normal may not be viable when placed into the reproductive tract. There may be the need for additional training of embryo transfer technicians to reduce the observed embryonic loss. An equally important need is for improved methods of morphological evaluation of embryos before transfer that are predictive of subsequent fertility.

Oocytes collected from the ovaries of slaughtered cows and matured/fertilized in vitro can be used to produce transferable embryos for dairy producers. The use of slaughterhouse oocytes has the advantage of scalability (i.e., many oocytes can be collected, matured, and fertilized) and this could possibly reduce the cost of embryos. The disadvantage of slaughterhouse oocytes is the incomplete genetic information of donor cattle.

In vitro gametogenesis is an emerging technology where gametes are generated from pluripotent stem cells and live offspring are produced (Aizawa et al., 2025). Yoshino et al. (2021) demonstrated the viability of this technique in mice. By combining fetal ovarian somatic-cell like cells (derived from pluripotent stem cells) with pluripotent stem cell-derived primordial germ-cell like cells, the authors were able to construct cumulus oocyte complexes (termed rOvaroids) that could be used for IVF and generate live offspring. The importance of this or similar techniques is that cells could come from any tissue or blood of an individual animal, dedifferentiated into stem cells (i.e., induced pluripotent stem cells) and used to create a renewable in vitro supply of oocytes from the same animal. Stem cells derived from either female (Yoshino et al., 2021) or male (Murakami et al., 2023) individuals could be used in this process, thus enabling oocyte production from both elite cows and elite sires. Targeted DNA methylation editing of imprinting control regions using clustered regularly interspaced short palindromic repeats (CRISPR)-based technology could enable the production of elite offspring from sperm cells derived from 2 adult sires or oocytes derived from 2 adult cows (Wei et al., 2025). Scientific advances in laboratory animals and humans will inform the work being done in cattle (de Bruin et al., 2025).

Blastocyst-like structures derived from pluripotent stem cells are called blastoids. Blastoids are important models for studying developmental biology of humans because their cells are derived from a cell line and avoid the ethical concerns of using a human embryo in research (Cheng et al., 2025). Studies of blastoids in cattle are advancing rapidly and create the possibility of a large supply of transferable embryos from cell lines derived from an individual animal. The embryos would be genetically identical (clones) to the parent animal. Ming et al. (2025) combined bovine embryonic pluripotent stems cells (bEPSC), bovine extraembryonic endoderm stem cells (bXEN), and bovine trophoblast stem cells (bTSC) to efficiently create blastoids (Ming et al., 2025). Their work stopped short of demonstrating the viability of the blastoids when transferred into recipient cattle. Earlier work by the same laboratory demonstrated IFNT production from blastoids transferred into recipient cattle (Pinzón-Arteaga et al., 2023). This work awaits the demonstration of live-born calves from blastoids and subsequent demonstration of scalable production and freezing of blastoids for on-farm applications. Stem cells matured into viable oocytes or used to construct viable blastocysts hold promise for efficient embryo production in the future. This new technology could entirely change the bovine embryo transfer industry and revolutionize dairy cattle breeding.

In summary, the future of technology applied to reproductive management of dairy cattle will consist of the refinement of existing technologies that include estrus detection, timed AI, sperm sorting, genomics, and pregnancy testing (Table 1). There will also be emerging technologies that are “barely achievable” today but could become “routine” tomorrow. Some important questions are unanswered and await future research (Table 2). The collective result of new knowledge and new technology will drive further improvements in genetics, fertility, and efficient reproduction in high-producing dairy cattle.
